# Predicting promoters targeted by TAL effectors in plant genomes: from dream to reality

**DOI:** 10.3389/fpls.2013.00333

**Published:** 2013-09-03

**Authors:** Laurent D. Noël, Nicolas Denancé, Boris Szurek

**Affiliations:** ^1^CNRS, Laboratoire des Interactions Plantes-Microorganismes (LIPM), UMR 2594Castanet-Tolosan, France; ^2^INRA, Laboratoire des Interactions Plantes-Microorganismes (LIPM), UMR 441Castanet-Tolosan, France; ^3^Institut de Recherche pour le Développement, UMR RPB IRD-CIRAD-UM2Montpellier Cedex 5, France

**Keywords:** TAL effectors, targets, prediction, *Xanthomonas*, susceptibility genes

## Introduction

Transcription Activator-Like (TAL) effectors from the plant pathogenic bacteria of the genus *Xanthomonas* are molecular weapons injected into eukaryotic cells to modulate the host transcriptome. Upon delivery, TAL effectors localize into the host cell nucleus and bind to the promoter of plant susceptibility (S) genes to activate their expression and thereby facilitate bacterial multiplication (Boch and Bonas, 2010; Schornack et al., 2013). In resistant plants, a few TAL effectors have been shown to bind to promoters of executor resistance (*R*) genes, resulting in localized cell death and preventing pathogen spread (reviewed in Doyle et al., 2013). Remarkably, TAL effectors harbor a novel type of DNA-binding domain with a unique modular architecture composed of 1.5–33.5 almost identical tandem repeats of 33–35 amino acids. Each repeat type specifies one or more bases through direct interaction with the second amino acid in a centrally located “Repeat Variable Diresidue” (RVD). The number and sequence of the RVDs across the whole repeat region of the TAL protein defines the DNA target. The code of DNA-binding specificity of *Xanthomonas* TAL effectors was inferred from experimental, computational and later on structural approaches (Boch et al., 2009; Moscou and Bogdanove, 2009; Deng et al., 2012; Mak et al., 2012). This new paradigm for protein-DNA interaction is now revolutionizing our perspectives for the understanding of TAL effectors roles during plant disease and defense since the identification of their plant targets is largely facilitated. A few algorithms are now available to predict *in silico* candidate genes of a given TAL effector. This *Opinion* gives an overview of the current tools and strategies that may be applied for finding targets of TAL effectors. We also raise limitations and pitfalls and emphasize what may be improved to gain in prediction accuracy. Finally, we also highlight several perspectives offered by these new tools.

## *In silico* prediction of TAL effectors targets

**Figure 1:**
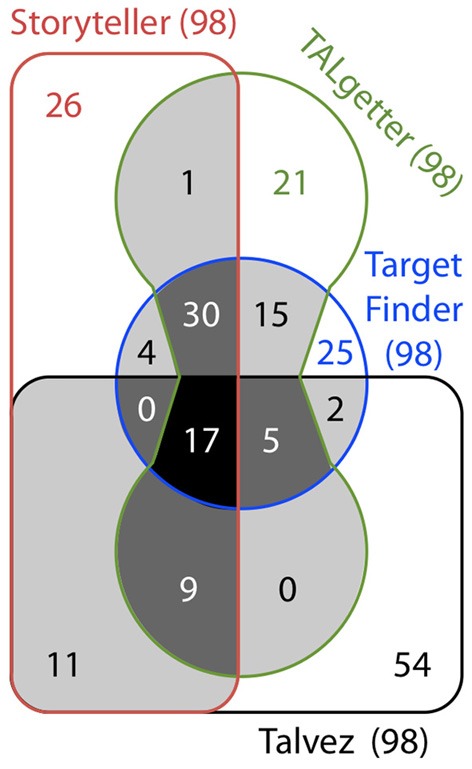
**Venn diagram representing the shared targets of Hax4 (GenBank AY993939; RVDs = NI HD HD NG NS NS NI HD NG NI NS NI NG NI NG) predicted in the Arabidopsis promoterome (1 kb upstream of the translation start site from the Col-0 ecotype genes in the TAIR annotation release 10) with the 4 currently available algorithms for TAL target prediction: *Storyteller* (parameters: rounds “10^5^”, noise “0.5”, noise-shape “hvaa-dependent,” max e-value “700,” minscore “2,” gap probability “10^−3^”), *TALgetter* (default parameters: “TALgetter long”), *Talvez* (version 3.1, parameters: pseudocounts “10^−5^”, minimum score “9”, number of reported TALEs “100” and position correction “19”) and *Target Finder* (TALE-NT 2.0, default parameters).** Shaded areas in light gray, dark gray and black indicate putative targets identified with 2, 3 or 4 prediction algorithms, respectively. Numbers in bracket indicate the total number of predicted targets for each prediction tool.

One major output of the modular TAL effector–DNA recognition code discovery is the possibility to predict through computer programs, the DNA binding sites of a TAL effector within a whole plant genome or promoterome (i.e., the sequences immediately upstream of the transcriptional start sites) of any sequenced organism. Four bio-informatic tools are currently available and enable to scan genomes for TAL effectors binding sites, rapidly providing users with lists of potential *S* or *R* targets. *Target Finder* from the TALE-NT 2.0 suite (https://tale-nt.cac.cornell.edu/, Doyle et al., 2012), *Talvez* (http://bioinfo.mpl.ird.fr/cgi-bin/talvez/talvez.cgi, Pérez-Quintero et al., 2013) and *Storyteller* (http://bioinfo-prod.mpl.ird.fr/xantho/tales, Pérez-Quintero et al., 2013) algorithms are available as web interface and/or standalone software. For these three examples, predictions rely on the use of a RVD-nucleotide association matrix based on known TAL effector–target pairs, to convert a sequence of RVDs of a given TAL effector into a positional weight matrix (PWM). These PWM are regularly updated based on novel experimental insights into TAL-DNA binding or the availability of experimentally confirmed TAL target sequences. *Target Finder* and *Talvez* both use the PWM to scan and score all possible binding sites in a promoter region with a log-likelihood function. In contrast, *Storyteller* uses this matrix to generate a set of possible binding sequences and takes the advantage of a faster pattern-search algorithm based on Hidden Markov models. Moreover, *Talvez* incorporates a position correction parameter, which enables to tolerate RVD-nucleotide mismatches toward the C-terminal end of RVD sequences and improves target sites prediction. Finally, *TALgetter* (http://galaxy.informatik.uni-halle.de; web interface or standalone) differs from the above-mentioned programs as it is based on a statistical model which parameters are estimated from training data computationally (Grau et al., 2013). Furthermore, *TALgetter* decodes the RVDs according to their binding specificity, but takes into account RVD “efficiency” or affinity, as reported by Streubel et al. (2012). Though these predictions yield a number of validated targets, we are still at early days. As an example, we used the Hax4 RVDs from *Xanthomonas campestris* pv. *campestris* strain Xca5 (Kay et al., 2005; Bolot et al., 2013) to mine for Arabidopsis targets in Col-0 promoterome using all 4 algorithms mentioned above. Among the 98 top targets identified by each algorithm, only 17 targets were predicted by all 4 algorithms and 51 by at least 3 algorithms (Figure 1). Although our knowledge on the efficiency (i.e., the percentage of validation of the predictions) of these bioinformatics tools remains poorly documented, significant differences exist between the four algorithms. Thus, combining predictions might help to reduce numbers of true targets for subsequent experimental validation. Yet, false negatives appear as the greatest threats in such approach since true biological targets could be missed this way. Further experimentally validation of TAL targets in different plant genomes are needed to improve the quality of the algorithms and move toward a higher confidence in the predictions.

## Predictions: caveats and possible improvements

Yet, our incomplete knowledge of the molecular mechanisms underlying the TAL-DNA interaction and subsequent transcriptional activation is a major limit of these predictions. Since the RVD-DNA code is somewhat degenerate, predictions for TALs with fewer repeats or rich in unspecific RVDs will yield significant amounts of false positive/false negative target sites especially when scrutinizing large plant genomes. Besides, our understanding of the relative contribution of each individual RVDs to the general protein affinity is still very scarce. Recently, it was shown both by reporter gene expression-based *in vivo* assays and/or biochemical studies that RVDs display different affinity to their favored nucleotides (Christian et al., 2012; Streubel et al., 2012; Meckler et al., 2013). These pioneer studies clearly point to the necessity of systematically evaluating the affinity of each individual most frequent but also rare RVDs for a given nucleotide. Other uncertainties include our incapacity to predict the effect of neighboring RVDs over the binding of a particular RVD, as well as the influence of the binding-site direct environment and the status of epigenetic marks. Finally, another source of inaccuracy in DNA-binding sites prediction deals with our difficulty to estimate TAL effectors tolerance for imperfect pairings which may vary depending on the type, position and context of the mismatch (Doyle et al., 2013). In the same line of idea, Meckler et al. (2013) recently showed that N-terminal RVDs contribute more to the overall DNA affinity than C-terminal RVDs. This result is corroborated by the analysis of Pérez-Quintero et al. (2013) showing from a set of well-characterized RVD-DNA interactions that perfect RVD-nucleotide pairing in TAL effectors N-terminal region (first 15–19 RVDs) probably determines for the most part the target DNA recognition and activity. Thus, mismatches in the C-terminal end of the repeat region generally appear to be better tolerated than in the N-terminal end. Altogether, this illustrates the fact that additional systematic experiments of both the binding affinity and specificity of each RVDs for their preferred nucleotides are required to optimize current predictive models, which would also gain in accuracy if trained with additional experimentally validated pairs of TALs and targets.

Independently of DNA-binding itself, transcriptional activation was strongly enhanced for TAL target sites in the −300 to +200 region relative to the transcriptional start site (TSS, Grau et al., 2013). Thus, proper structural annotation of genomes including RNAseq-based or EST based annotation of TSS should greatly enhance the quality of the predictions. Though not formally included in the current algorithms, filtering manually for putative target sites close to transcriptional or translational start sites is advisable.

## *In silico* or wet lab?: probably both!

What comes up as an obvious and promising strategy is the use of experimental data to identify new targets. Recently, *Bs4C* executor target was identified solely based on a thoroughly designed RNAseq approach in pepper. The *X. axonopodis* pv. *vesicatoria* AvrBs4 TAL effector target was pinned down to a single promoter to which direct binding was demonstrated (Strauss et al., 2012). Yet, Q-RT-PCR can also be used to confirm predicted targets and may be a cheap shortcut when whole transcriptome profiling (micro-arrays or RNAseq) is not an option: prediction algorithms yield a number of true positives. For instance, 21 TAL targets predicted by *Target Finder* in the rice genome for 14 presumably *X. oryzae* TALs predicted could be verified experimentally (Doyle et al., 2012). As already shown in several studies, comparing the transcriptome of plants challenged with *Xanthomonas* strains carrying a TAL effector of interest vs. a strain defective for that particular *tal* gene or mock inoculation, produces lists of up-regulated genes which are enriched for direct *S* or *R* targets of the TAL effector under study (Yang et al., 2006; Sugio et al., 2007; Yu et al., 2011). Hence, one strategy for evaluating the validity of computationally predicted virulence targets is certainly to benchmark them against TAL effector-dependent profiling experiments, as successfully applied to assess the validity of *TALgetter* (Grau et al., 2013) and *Talvez* (Pérez-Quintero et al., 2013). Nevertheless, a main concern in the overall process is due to the difficulty of discriminating direct and biologically relevant TAL targets from direct and biologically irrelevant TAL targets or secondary/indirect targets. Indeed, off-targets can be found predicted and induced, inherently to the degeneracy of the TAL effector—DNA recognition code and as exemplified by the *X*. *oryzae* pv. *oryzae* TAL effector AvrXa7 which induces both the expression of the well-characterized susceptibility gene *OsSWEET14* and a gene coding for a retrotransposon (Li et al., 2012). Secondary indirect targets might be induced independently of the presence of the *tal* gene or as a result of the induction of a TAL direct target. The use of a cycloheximide treatment can help to identify genes which expression does not directly result from TAL activity. One alternative way to counter select off-targets may be to favor candidate targets subjected to functional convergence events, as illustrated for the rice susceptibility gene *OsSWEET14*, which was found to be activated by 4 different TAL effectors originating from 4 different strains of *Xoo* and binding to 3 different target sites in the *OsSWEET14* promoter. Upon the analysis of the *Xoo* TAL repertoire for which targets where predicted and compared to publicly available expression data, several instances of functional convergence between different strains could be demonstrated (Pérez-Quintero et al., 2013).

## Perspectives: TAL *S* targets as new tools to decipher host specialization of *xanthomonas* species?

Despite recent breakthrough in TAL effectors biology, the contribution of TAL targets in promoting susceptibility is yet poorly understood. This is particularly true considering the diversity of the *Xanthomonas* genus (27 species and more than 100 pathovars), of the diseases caused on more than 400 different host plants and of the corresponding TAL repertoire (none to 26 TAL copies per strain). The discovery of the *Xanthomonas* TALome is a major task which is seriously hindered by the fact that current sequencing technologies and genome assembly pipelines cannot properly assemble the highly repetitive TAL DNA sequences from whole genome shotgun sequencing data. Also, our knowledge about the relative contribution of TAL effectors to pathogenicity in strains containing multiple *tal* genes is limited to a “happy few” pathosystems such as *X. axonopodis* pv. *vesicatoria*, *X. citri* pv. *citri*, *X. oryzae* pv. *oryzae*, *X*. *axonopodis* pv. *malvacearum* and *X*. *axonopodis* pv. *manihotis*. Revealing the susceptibility genes involved in these processes will be key to deciphering as many potentially unique disease scenarios and represent unprecedented means to access a wealth of information and dissecting the molecular executors of susceptibility. In fact, identifying major virulence TAL effectors of well-studied and more exotic *Xanthomonas* pathovars and fishing their targets offers a unique strategy to understand what may drive host specialization in a species level.

## Concluding remarks

As time passes, experimental data will accumulate and help to refine the prediction algorithms. Yet, the most challenging aspect remains the biology of the *Xanthomonas*/plant interaction. During the co-evolution process, bacteria have selected a TAL repertoire to adapt to the diversity of natural hosts and the selection of novel crop species by humans. The latter might be the reason why some strains of the rice pathogens *Xoo* and *Xoc* have so many TALs (up to 26) (Schornack et al., 2013). Therefore, the choice of the right host plant genotype to find the genuine TAL targets is critical. One will always find a target for a TAL in any plant or even animal genome. The experimental validation of target gene induction or direct TAL-binding to the promoter still does not indicate that the right biological system was studied. If the *tal* gene studied contributes significantly to the pathogenicity on the selected plant genotype, one has the chance to find important *S* genes. Yet, in nature, the contribution of many TAL effectors to disease development will be subtle and dependent on the plant genotype. This means that in the future, *Xanthomonas* and diseased hosts should be sampled together in epidemics to advance in the identification of genuine TAL targets and in our understanding of *Xanthomonas* virulence strategies. Combining pathosystems isolated from natural epidemics with *in silico*, genomic and transcriptomic approaches are certainly the way to go in the next decade. These approaches should yield a large number of targets which contribute quantitatively to susceptibility and resistance for marker-assisted breeding in important crop species.
